# Evolution of the human hip. Part 1: the osseous framework

**DOI:** 10.1093/jhps/hnu013

**Published:** 2014-10-28

**Authors:** Tom Hogervorst, Evie E. Vereecke

**Affiliations:** ^1^Haga Hospital, Sportlaan 600, 2566MJ The Hague, Netherlands and ^2^Department of Development & Regeneration @ Kulak, KU Leuven, Etienne Sabbelaan 53, 8500 Kortrijk, Belgium.

## Abstract

Extensive osseous adaptations of the lumbar spine, pelvis, hip and femur characterize the emergence of the human bipedal gait with its ‘double extension’ of the lumbar spine and hip. To accommodate lumbar lordosis, the pelvis was ‘compacted’, becoming wider and shorter, as compared with the non-human apes. The hip joint acquired a much more extended position, which can be seen in a broader evolutionary context of verticalization of limbs. When loaded in a predominantly vertical position, the femur can be built lighter and longer than when it is loaded more horizontally because bending moments are smaller. Extension of the hip joint together with elongation of the femur increases effective leg length, and hence stride length, which improves energy efficiency. At the hip joint itself, the shift of the hip’s default working range to a more extended position influences concavity at the head–neck junction and femoral neck anteversion.

## INTRODUCTION

Compared to other mammals, the human hip has several unique features. While *in* *utero* the human limb is hyperflexed, the default loading position shifts close to the hip (and knee) extension limit with the development of upright gait. Other mammals, including the non-human apes (gibbon, chimpanzee, bonobo, gorilla and orangutan), have a ‘mid-flex’ hip position as their default. In fact, no mammal has a habitual extended hip position like humans do. Even other habitual bipeds, such as kangaroos, have a flexed hip position. This peculiar extended position of the hip in the obligate bipedal human required extensive osseous adaptations, and, not surprisingly, these went hand in hand with muscular changes.

Around the world, hip surgeons pay increasing attention to the extra-articular tissues. Muscle preserving techniques, aiming to avoid tenotomy altogether, are used increasingly for arthrotomy and arthroplasty. Arthroscopic surgeons now perform repairs of gluteus medius, rectus femoris and hamstrings, iliopsoas tenotomies and decompressions, trochanteric bursectomies, sciatic, obturator and pudendal neurolyses, lengthening of the iliotibial tract, etc. Understanding how the muscles and other soft tissues have evolved around the hip in the human lineage could be helpful to better understand injury and overuse patterns. In two papers, we explore the consequences of the extended stance on the configuration of the hip joint and surrounding soft tissue. Part 1 explores the osseous adaptations of the lumbo-pelvic-hip complex, whereas part 2 examines the changes in the muscles and tendons that accompanied the transition to permanent upright gait, focussing on hip extensors, flexors and abductors.

## OSSEOUS ADAPTATIONS OF FEMUR AND PELVIS

### Verticalization

**Figure 1.**
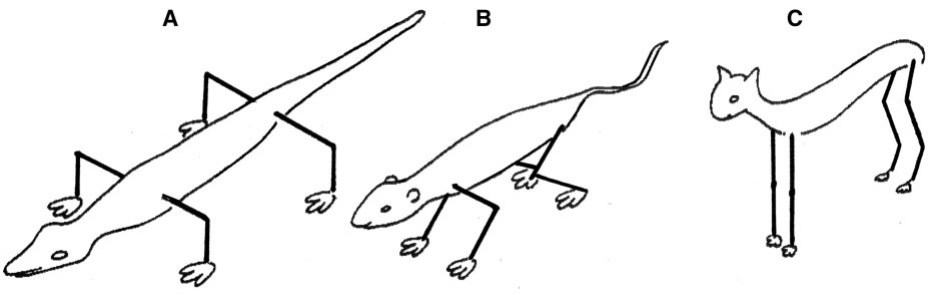
Steps in verticalization of limbs, placing the limbs under the trunk. (A) Reptile, (B) non-cursorial mammal and (C) cursorial mammal, from [6].

**Figure 2.**
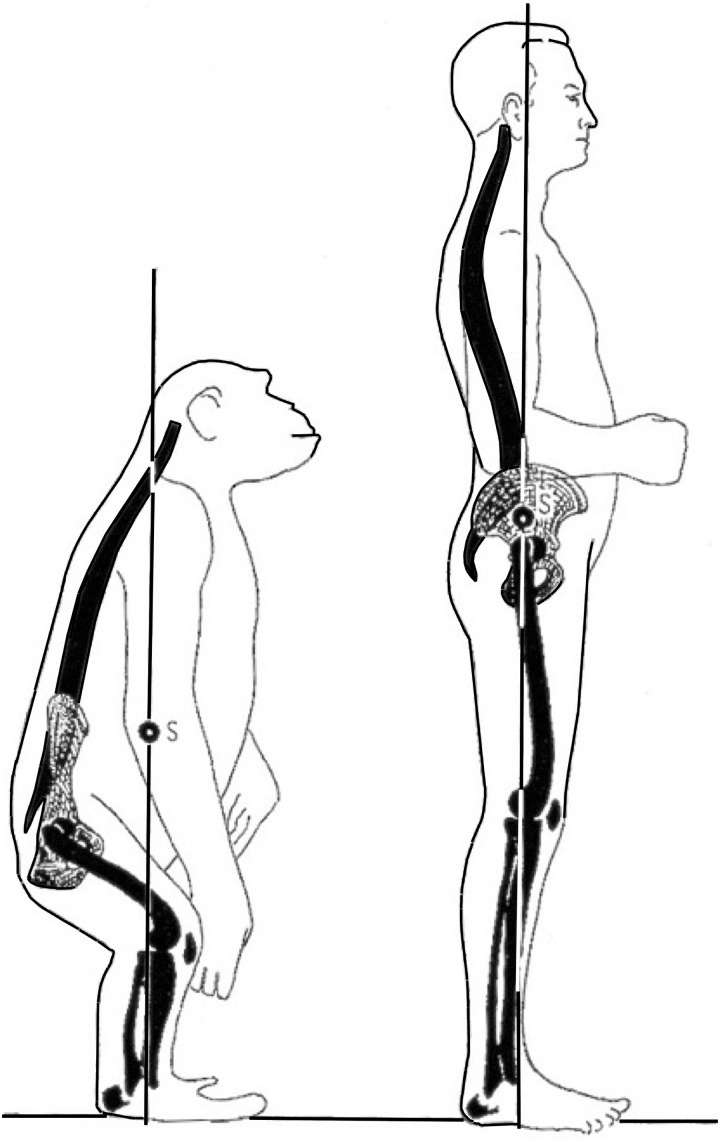
Double extension of the hip and spine: a vertical femur and lumbar lordosis (from [10]).

**Figure 3.**
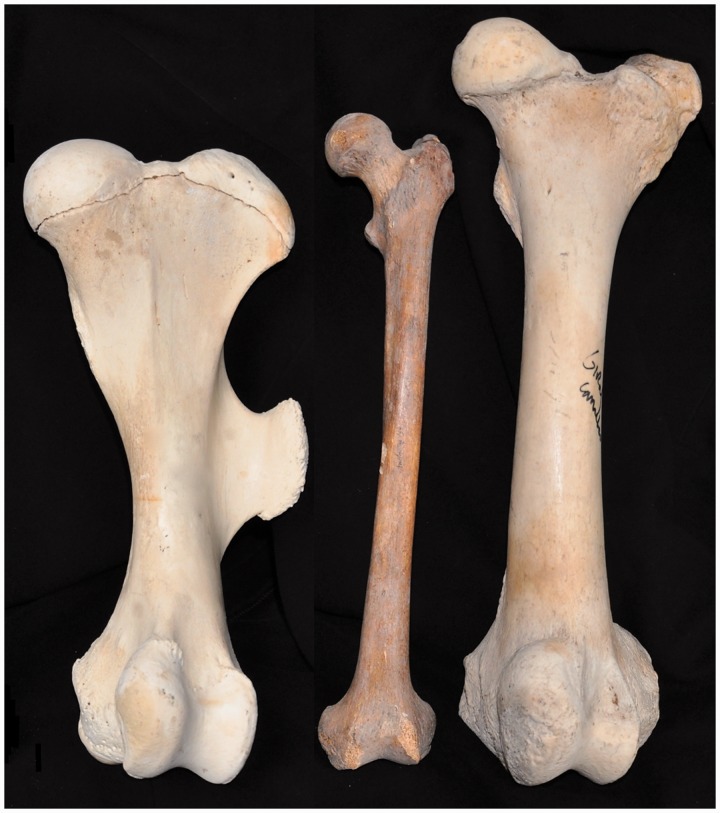
A long femur indeed: anterior view of a femur of a rhinoceros (graviportal, left), human and giraffe (cursorial, right). Scale is identical for all three femora.

When loads increase, limbs tend to rearrange towards a more vertical orientation. Such verticalization decreases the work for the muscles that have to counteract gravity to support the trunk. For example, the hip in a quadruped such as a horse experiences a flexion moment during gait when the hoof contacts the ground. This flexion moment is counteracted by the hip extensors, but in a more vertical femur far less work is required to do so (because the flexion moment is proportional to the horizontal component of the femur lever arm. This is analogous to a structure supported by a vertical beam versus an angled one). Three examples of such vertical rearrangement can be seen in (i) the change in the transverse plane from a sprawling to more erect limb position from reptiles to mammals (Fig. 1), (ii) the shortening and verticalization of the femoral neck in graviportals such as elephants and rhinos (Fig. 3) and finally, and of particular relevance here, (iii) the verticalization of the human femur for obligate bipedalism (Figs 2 and 3). This verticalization of the human femur is realized by hip extension and optimizes an energy efficient gait because it reduces the work required by hip extensors. In quadrupeds, a more horizontally oriented femur requires the gluteus medius and hamstrings to counteract hip flexion at hoof/paw strike. These muscles also generate the power to extend the hip at push-off, and they are massive. In the horse, the biceps femoris, for example, is second only to the gluteus medius in hindlimb muscle mass, and these two muscles comprise 34% of total hindlimb muscle mass [1]. In this set-up, a long ‘horizontal’ femur would require a lot of work on hoof strike, while in contrast either a short ‘horizontal’ femur or a ‘vertical’ femur greatly reduces the work required on heel strike. While flexion moments are relatively small at the human hip, they can be substantial at the human knee, for example, at heel strike or in stair walking. The quadriceps counteracts these knee flexion moments and this helps explain why humans have roughly twice the quadriceps volume compared with hamstrings (Q:H ratio), the reverse of what is seen in quadrupeds [2, 3]. We found that chimpanzees and gorillas have a roughly 1:1 Q:H ratio, whereas gibbons approach the human condition of 2:1, probably because of the importance of leaping in these Asian apes [4, 5].

### Double extension

With the acquisition of an upright gait, not only did the hip joint extend, but the (lumbar) spine as well, hence a ‘double extension’ of both spine and hip evolved in the human lineage. The resulting human bipedal gait features a unique combination of pendular limb motion and an orthograde spine, not seen in other vertebrates [7] [penguins do have an erect spine, but their waddling (instead of pendular) gait carries a high energy cost].

The relatively long lumbar spine of humans, together with the broad iliac blades, gives the lumbar region a high mobility and facilitates the adoption of a lumbar lordosis, which is essential for an efficient upright gait [8]. Unlike humans, the African apes have a short and stiff lumbar spine, which positions the centre of mass in front of the hip during a bent-hip, bent-knee bipedal gait (Fig. 2). This flexed position of hip and knee leads to an unfavourable leverage at these joints and, hence, a high locomotor cost [9]. For the acquisition of habitual bipedal walking, and certainly for bipedal running, the ability to develop a lumbar lordosis has likely been crucial in our early hominin ancestors. The drawback of having a relatively long and mobile lumbar spine is its proneness to injury.

### Long femur

Compared to other mammals, humans have long femora (Fig. 3). While quadrupedal runners, such as the dog, horse or giraffe (Fig. 3), have a short and stout femur, humans have a relatively slender and long femur. This can be explained by the extended position of the hip in humans which places the human femur in a more vertical working range and reduces bending moments on the femur compared to femora with a more horizontal working range. Having a long, vertical femur becomes advantageous in bipedal gait because it increases effective leg length, as such increasing stride length (Fig. 2) [11–13]. Other features that allow an effective leg lengthening are pelvic rotation and tilt (in the transverse and frontal plane), full extension of the knee and ankle plantarflexion at push-off [14]. Other features found in modern humans that contribute to the efficiency of bipedalism are distal femoral valgus to align the knee in the leg’s mechanical axis (‘bicondylar angle’ [15]), a well-developed Achilles tendon to store and release elastic energy [16], a foot that came to function as a lever with a spring (the plantar aponeurosis [17]) and an adducted, robust [18] hallux for push-off. The non-human apes have very different feet, with a widely abducted hallux, that function much more as a flexible grasping tool than a lever [19, 20].

### Short and wide pelvis

**Figure 4.**
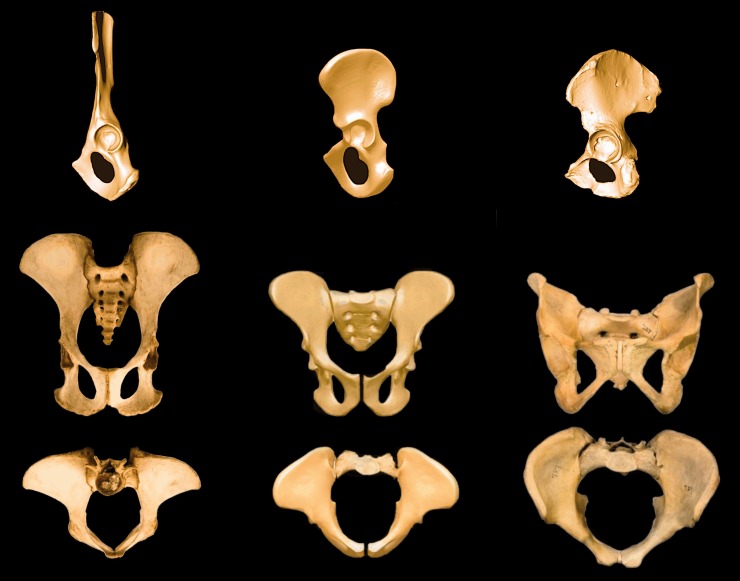
The Ardipithecus pelvis at 4.4 mya was already more human- than chimpanzee-like. Three pelves in lateral (top row), anteroposterior and axial views (bottom row). From left to right: Chimpanzee (*Pan troglodytes*), *Ardipithecus ramidus* of 4.4 mya and *Homo sapiens* (*A. ramidus* adapted from [24], with permission).

The pelvis has undergone dramatic changes in both shape and orientation during the course of human evolution [21]. Important changes are related to (i) a ventral expansion of the ilium, resulting in a shift from an essentially 2D ilium to a 3D ilium with wide flared blades and (ii) a dorsal projection of the ischial tuberosities (Fig. 4). These osteological changes are associated with a repositioning of musculature, and therefore changes in muscle function. The 3D ilium of modern humans results in the gluteus maximus becoming a powerful hip extensor, which is particularly important during bipedal running [22]. The gluteus medius and minimus shifted to being primary hip abductors, which are important for stabilization of the pelvis and spine in the frontal plane during bipedal walking and running. Due to the flaring of the human ilium, the posterior superior iliac spines receive a more posterior position, resulting in a more effective leverage for the extensor muscles of the spine which are important for bipedal locomotion and carrying [23]. As will be discussed in detail in Part 2, the dorsal orientation of the ischial tuberosities affects the functionality of the hip extensors, i.e. the hamstrings.

### The human hip: open anterior*,* high concavity posterior

**Figure 5.**
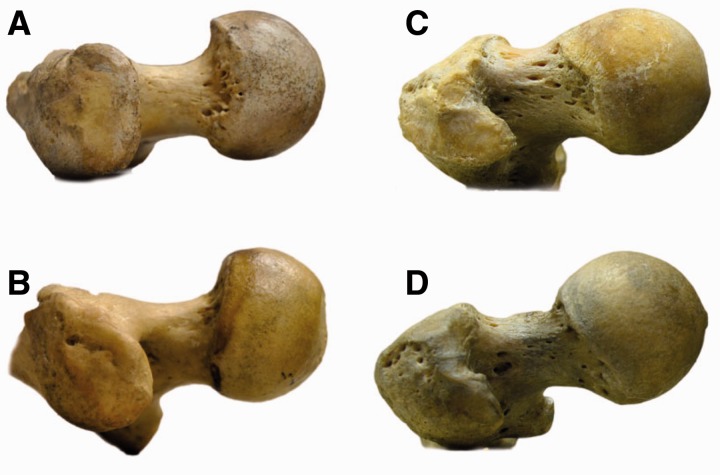
Concavity in non-human apes and humans. (A) gorilla, (C) chimpanzee, (C and D) human. The non-human apes uniformly have large concavity and more so anteriorly than posteriorly. Some humans (C) have only small concavity anteriorly, others have larger anterior concavity (d), but virtually all humans have large concavity posteriorly (C, D). View is perpendicular to the superior femoral neck, from [26], with permission.

Extending the hip joint means the hip is now ‘open’ anteriorly. In most mammals, the resting or neutral position of the hip can be inferred by aligning the articular cartilage margins of femoral head and acetabulum. But in a supine human, with the hip resting in a neutral (0° extension) position, a substantial portion of the femoral head is uncovered anteriorly. Conversely, posteriorly part of the femoral head cartilage and part of the head–neck junction lie within the acetabulum. For further extension, ‘posterior concavity’ is important (Fig. 5). Concavity is a compound parameter determined by femoral head sphericity, head–neck offset and the position of the femoral head on the neck. It can be seen as the femoral-sided osseous determinant of an impingement-free range of hip motion. A cam morphotype femur has low concavity in the anterior and superior aspect of the head–neck junction (Fig. 5c). But this morphotype, although associated to development of osteoarthritis [25], is likely not important in an evolutionary sense, i.e. it has no direct effect on evolutionary fitness [26]. Conversely, low posteroinferior concavity is virtually unknown [27]. The reason for this could be that loss of posteroinferior concavity would have a real effect on evolutionary fitness because it would prevent normal gait development by limiting hip extension for toe-off. In an evolutionary sense, loss of anterosuperior concavity is tolerated well, but posteroinferior concavity appears critical for normal gait development and performance.

Thus, in femoroacetabular impingement, the femoral-sided low concavity is always anterosuperior and very rarely posteroinferior. This is the reverse situation as seen in quadrupedal runners. Quadrupeds, such as the horse, have low concavity at the posterosuperior aspect of the hip, high concavity anteriorly. We postulate that this might be related to the region of the head–neck junction that absorbs peak loads at hoof strike in running. Due to the double extension and upright versus horizontal trunk axis (orthograde versus pronograde), this region has shifted ∼ 90° anteriorly at heel strike in the human hip [28]. The non-human apes are yet another story in this concept. They do not run or walk long distances bipedally [29], but do need a large range of hip motion in climbing and clambering, facilitated by circumferential high concavity at the head–neck junction [26].

### Anteversion and neck-shaft angle: 3D parameters for a 3D joint

Concluding the osseous adaptations associated with upright bipedal gait, we speculate on the functional significance of proximal femoral anteversion and neck-shaft angle. Using an identical measurement method to compare a sample of 375 human and 211 African ape femora, we found both anteversion and neck-shaft angle around 5° higher in humans than the African apes [26]. The Asian orangutan differs in that it has retroversion and a higher neck-shaft angle (Table I). Femoral version is quite variable in both humans and non-human apes, and in the non-human apes retroversion is not an exception.

**Table I.** Version and neck-shaft angle in non-human apes and humans, measured according to [27]

	Gorilla^a^	Pan^a^	Orangutan^b^	Homo^c^
*n*	92	119	13	375
Neck version	4.6 (7.0)	5.3 (6.9)	−1.6 (6.9)	9.7 (9.3)
Neck-shaft angle	123.0 (5.7)	125.6 (5.2)	137.2 (4.5)	129.2 (6.3)

Mean values are given with (standard deviation). Negative values denote retroversion. Data from ^a^[26], ^b^[28] and ^c^[27].

Pauwels *et al.* [30] originally postulated that unequal pressure on one side of the growth plate would accelerate growth on that side, until the compression was equal again across the growth plate [30]. In other words, during growth and development, the femoral head growth plate remains approximately perpendicular to the habitual angle of the hip joint reaction force. Accordingly, the neck-shaft angle is around 150° in newborn humans [15], but decreases when abductor forces develop with bipedal gait, making the joint reaction force more horizontal. When the abductor forces do not fully develop, as may happen in cerebral palsy (CP) or developmental hip dysplasia (DDH), we find the neck-shaft angle remains high or increases further. Pauwels based his idea primarily on uniaxial forces, but his concepts were confirmed by 3D analysis for DDH and CP [31]. This situation is analogous to the knee, where the epiphysis is perpendicular to the femoral shaft in newborns, but acquires a valgus angle when bipedal gait develops. This aligns the distal growth plate of the femur perpendicular to the predominant joint reaction forces, reflected in the frontal plane by the bicondylar (valgus) angle and in the sagittal plane by the slope angle of the tibial plateau [32].

Clinical conditions such as CP and DDH underscore the plasticity of the proximal femur as reflected in neck-shaft [33] and anteversion angles. Somewhat less pronounced effects on neck-shaft angle are known for the loading history of the hip as related to culture and climatic region. Cultures with higher hip loading such as hunter-gatherers [34, 35], and colder climates [34] are associated with lower neck-shaft angle, and the latter has been suggested to explain the lower neck-shaft angle of Neanderthals [36]. Furthermore, loading history was found to influence morphogenesis of the head–neck junction, with adolescents involved in strenuous sports having lower concavity than controls [37, 38]. These findings bring up the question of the relation between these three parameters (version, neck-shaft angle and concavity), but this has yet to be rigorously examined.

From an evolutionary perspective, in a ‘horizontal’ femur of a quadruped, anteversion better aligns the femoral neck with the forces at heel strike [39]. Although this effect of anteversion is diminished (but not absent) in the extended human hip, it is partly taken over by the neck-shaft angle, as both angles are a planar expression of a 3D relation between the femoral shaft and neck. Both the neck-shaft angle and anteversion (to a certain limit) help to align the proximal femoral epiphysis more perpendicular to the impact forces of heel strike in bipedal gait. Perhaps then, low neck-shaft angle and/or low femoral anteversion or retroversion is related to the likelihood of developing a coxa recta (cam morphotype). One earlier study indeed reported low prevalence of coxa recta in populations with higher anteversion (and similar neck-shaft angle) (Hoaglund and Low 1980). More recent studies in small patient groups show lower anteversion in cam hips versus controls [40], or no difference [41], but neither study examined both version and neck-shaft angle.

It may appear that the human hip evolved to higher anteversion and neck-shaft angle from that of the non-human apes, but this view would disregard the fact the non-human ape hip is not a precursor or ancestral version to the human hip. The hips of the extant non-human apes have gone through their own development in several key traits [42]. One of these appears to be a decrease in anteversion. For the non-human apes, low anteversion or retroversion is likely a useful adaptation for tree climbing as it allows them to hold on to substrates with their feet, also when these are located behind them. Although anteversion has not been measured in many mammal species, it appears most mammals, including modern humans, have higher anteversion than the non-human apes [39]. Therefore, regarding version, it is the non-human ape hip that appears remarkable, rather than the human hip.

## CONCLUSION

Although the debate on evolutionary mechanisms leading to a habitual upright bipedal gait is ongoing, once en route to bipedality, evolutionary mechanisms likely favoured energetic efficiency of the (early) human hip to drive bipedal gait, over maximum power production as needed for climbing in the non-human apes. As a walker/runner, humans do not stand out for maximal power or top speed, but perform quite well in endurance [18]. This is because efficient mechanics, such as the vertical femur and double extension, are combined with a breathing mechanism that has decoupled stride from inspiration [43]. Quadrupeds have a 1:1 ratio of gait and breathing cycles, but bipedal human runners can decouple their breathing and gait cycles [43]. Furthermore, sweating increases thermal regulation in endurance running [18].

By default, the extensive osseous adaptations described earlier were accompanied by soft-tissue and muscular changes. In part 2 of this article, we will explore the muscular requirements and consequences of running and sprinting, and categorize the soft-tissue consequences of surgical approaches to the hip.

## CONFLICT OF INTEREST STATEMENT

None declared.
